# Revision and Update of the Consensus Definitions of Invasive Fungal Disease From the European Organization for Research and Treatment of Cancer and the Mycoses Study Group Education and Research Consortium

**DOI:** 10.1093/cid/ciz1008

**Published:** 2019-12-05

**Authors:** J Peter Donnelly, Sharon C Chen, Carol A Kauffman, William J Steinbach, John W Baddley, Paul E Verweij, Cornelius J Clancy, John R Wingard, Shawn R Lockhart, Andreas H Groll, Tania C Sorrell, Matteo Bassetti, Hamdi Akan, Barbara D Alexander, David Andes, Elie Azoulay, Ralf Bialek, Robert W Bradsher, Stephane Bretagne, Thierry Calandra, Angela M Caliendo, Elio Castagnola, Mario Cruciani, Manuel Cuenca-Estrella, Catherine F Decker, Sujal R Desai, Brian Fisher, Thomas Harrison, Claus Peter Heussel, Henrik E Jensen, Christopher C Kibbler, Dimitrios P Kontoyiannis, Bart-Jan Kullberg, Katrien Lagrou, Frédéric Lamoth, Thomas Lehrnbecher, Jurgen Loeffler, Olivier Lortholary, Johan Maertens, Oscar Marchetti, Kieren A Marr, Henry Masur, Jacques F Meis, C Orla Morrisey, Marcio Nucci, Luis Ostrosky-Zeichner, Livio Pagano, Thomas F Patterson, John R Perfect, Zdenek Racil, Emmanuel Roilides, Marcus Ruhnke, Cornelia Schaefer Prokop, Shmuel Shoham, Monica A Slavin, David A Stevens, George R Thompson, Jose A Vazquez, Claudio Viscoli, Thomas J Walsh, Adilia Warris, L Joseph Wheat, P Lewis White, Theoklis E Zaoutis, Peter G Pappas

**Affiliations:** 1 Department of Hematology, Radboudumc, Nijmegen, The Netherlands; 2 Centre for Infectious Diseases and Microbiology, Laboratory Services, Institute of Clinical Pathology and Medical Research, Westmead Hospital, University of Sydney, Sydney, Australia; 3 Division of Infectious Diseases, University of Michigan, VA Ann Arbor Healthcare System, Ann Arbor, Michigan, USA; 4 Department of Pediatrics, Duke University Medical Center, Durham, North Carolina, USA; 5 Department of Medicine, Division of Infectious Diseases, University of Alabama at Birmingham, Birmingham, Alabama, USA; 6 Center of Expertise in Mycology Radboudumc/CWZ, Nijmegen, The Netherlands; 7 University of Pittsburgh, Pittsburg, Pennsylvania, USA; 8 Department of Medicine, University of Florida College of Medicine, Gainesville, Florida, USA; 9 Mycotic Diseases Branch, Centers for Disease Control and Prevention, Atlanta, Georgia, USA; 10 Infectious Disease Research Program, Center for Bone Marrow Transplantation and Department of Pediatric Hematology and Oncology University Children’s Hospital, Münster, Germany; 11 University of Sydney, Marie Bashir Institute for Infectious Diseases & Biosecurity, University of Sydney School of Medicine Faculty of Medicine and Health, Westmead Institute for Centre for Infectious Diseases and Microbiology, Western Sydney Local Health District, Sydney, Australia; 12 Infectious Disease Clinic, Department of Medicine University of Udine and Department of Health Sciences, DISSAL, University of Genoa, Genoa, Italy; 13 Ankara University, Faculty of Medicine, Cebeci Campus, Hematology Clinical Research Unit, Ankara, Turkey; 14 Department of Medicine and Division of Infectious Diseases, Duke University Medical Center, Durham, North Carolina, USA; 15 Division of Infectious Diseases, Departments of Medicine, Microbiology and Immunology School of Medicine and Public Health and School of Pharmacy, University of Wisconsin, Madison, Wisconsin, USA; 16 Médicine Intensive et Réanimation Hôpital Saint-Louis, APHP, Université Paris Diderot, Paris, France; 17 Molecular Diagnostics of Infectious Diseases, Microbiology, LADR Zentrallabor Dr. Kramer & Kollegen, Geesthacht, Germany; 18 Division of Infectious Diseases, University of Arkansas for Medical Sciences, Little Rock, Arkansas, USA; 19 Institut Pasteur, Molecular Mycology Unit, CNRS UMR2000, Mycology Laboratory, Saint-Louis Hospital, Assistance Publique-Hôpitaux de Paris, Université de Paris, Paris, France; 20 Infectious Diseases Service, Department of Medicine, Lausanne University Hospital and University of Lausanne, Lausanne, Switzerland; 21 Department of Medicine, Alpert Warren Medical School of Brown University, Providence, Rhode Island, USA; 22 Infectious Diseases Unit, IRCCS Istituto Giannina Gaslini, Genova, Italy; 23 Infectious Diseases Unit, G. Fracastoro Hospital, San Bonifacio, Verona, Italy; 24 Spanish National Centre for Microbiology, Instituto de Salud Carlos III, Madrid, Spain; 25 Infectious Diseases Division, Department of Medicine, Uniformed Services University of the Health Sciences, Bethesda, Maryland, USA; 26 National Heart & Lung Institute, Imperial College London, the Royal Brompton & Harefield NHS Foundation Trust, London, UK; 27 Pediatric Infectious Diseases Division at the Children’s Hospital of Philadelphia, Philadelphia, Pennsylvania, USA; 28 Centre for Global Health, Institute for Infection and Immunity, St Georges University of London, London, UK; 29 Diagnostic and Interventional Radiology, University Hospital Heidelberg, Translational Lung Research Center and Diagnostic and Interventional Radiology with Nuclear Medicine, Thoraxklinik Heidelberg, Heidelberg, Germany; 30 Faculty of Health and Medical Sciences, University of Copenhagen, Copenhagen, Denmark; 31 Centre for Medical Microbiology, University College London, London, UK; 32 UT MD Anderson Cancer Center, Houston, Texas, USA; 33 Radboud Center for Infectious Diseases and Department of Medicine, Radboudumc, Nijmegen, The Netherlands; 34 Department of Microbiology, Immunology and Transplantation and Department of Laboratory Medicine and National Reference Centre for Mycosis, University Hospitals Leuven, Leuven, Belgium; 35 Infectious Diseases Service, Department of Medicine and Institute of Microbiology, Lausanne University Hospital and University of Lausanne, Lausanne, Switzerland; 36 Pediatric Hematology and Oncology, Hospital for Children and Adolescents, University of Frankfurt, Frankfurt, Germany; 37 Molecular Biology and Infection, Medical Hospital II, WÜ4i, University Hospital Würzburg, Würzburg, Germany; 38 Paris University, Necker Pasteur Center for Infectious Diseases and Tropical Medicine, IHU Imagine & Institut Pasteur, Molecular Mycology Unit, CNRS UMR 2000, Paris, France; 39 Department of Hematology, University Hospitals Leuven, Leuven, Belgium; 62 Department of Microbiology, Immunology and Transplantation, K.U. Leuven, Leuven, Belgium; 40 Department of Medicine, Division of Infectious Diseases, Johns Hopkins University School; 41 Critical Care Medicine Department NIH-Clinical Center, Bethesda, Maryland, USA; 42 Department of Medical Microbiology and Infectious Diseases and Centre of Expertise in Mycology Radboudumc/Canisius Wilhelmina Hospital, Nijmegen, The Netherlands; 43 Alfred Health and Monash University, Melbourne, Australia; 44 Department of Internal Medicine, Universidade Federal do Rio de Janeiro, Rio de Janeiro, Brazil; 45 Division of Infectious Diseases, McGovern Medical School, Houston, Texas, USA; 46 Istituto di Ematologia, Università Cattolica S. Cuore, Rome, Italy; 47 UT Health San Antonio and South Texas Veterans Health Care System, San Antonio, Texas, USA; 48 Department of Internal Medicine–Hematology and Oncology, Masaryk University and University Hospital Brno, Brno, Czech Republic; 49 Infectious Diseases Unit, 3rd Department of Pediatrics, Faculty of Medicine, Aristotle University School of Health Sciences, Hippokration General Hospital, Thessaloniki, Greece; 50 Department of Hematology & Oncology, Lukas Hospital, Buende, Germany; 51 Meander Medical Center Amersfoort and Radiology, Radboud University Medical Center, Nijmegen, The Netherlands; 52 Department of Infectious Diseases, Peter MacCallum Cancer Center and the National Centre for Infections in Cancer, The University of Melbourne, Melbourne, Victoria, Australia; 53 Division of Infectious Diseases and Geographic Medicine, Stanford University Medical School, Stanford, California; 64 California Institute for Medical Research, San Jose, California, USA; 54 Department of Internal Medicine, Division of Infectious Diseases, University of California Davis Medical Center, Sacramento, California, USA; 55 Division of Infectious Diseases, Medical College of Georgia/Augusta University, Augusta, Georgia, USA; 56 Division of Infectious Disease, University of Genova and San Martino University Hospital, Genova, Italy; 57 Weill Cornell Medicine of Cornell University, Departments of Medicine, Pediatrics, Microbiology & Immunology, New York, New York, USA; 58 MRC Centre for Medical Mycology at the University of Aberdeen, Aberdeen, UK; 59 MiraVista Diagnostics, Indianapolis, Indiana, USA; 60 Public Health Wales Mycology Reference Laboratory, University Hospital of Wales, Heath Park, Cardiff, UK; 61 Perelman School of Medicine at the University of Pennsylvania, Children’s Hospital of Philadelphia and Roberts Center for Pediatric Research, Philadelphia, Pennsylvania, USA

**Keywords:** consensus, definitions, invasive fungal diseases, diagnosis, research

## Abstract

**Background:**

Invasive fungal diseases (IFDs) remain important causes of morbidity and mortality. The consensus definitions of the Infectious Diseases Group of the European Organization for Research and Treatment of Cancer and the Mycoses Study Group have been of immense value to researchers who conduct clinical trials of antifungals, assess diagnostic tests, and undertake epidemiologic studies. However, their utility has not extended beyond patients with cancer or recipients of stem cell or solid organ transplants. With newer diagnostic techniques available, it was clear that an update of these definitions was essential.

**Methods:**

To achieve this, 10 working groups looked closely at imaging, laboratory diagnosis, and special populations at risk of IFD. A final version of the manuscript was agreed upon after the groups’ findings were presented at a scientific symposium and after a 3-month period for public comment. There were several rounds of discussion before a final version of the manuscript was approved.

**Results:**

There is no change in the classifications of “proven,” “probable,” and “possible” IFD, although the definition of “probable” has been expanded and the scope of the category “possible” has been diminished. The category of proven IFD can apply to any patient, regardless of whether the patient is immunocompromised. The probable and possible categories are proposed for immunocompromised patients only, except for endemic mycoses.

**Conclusions:**

These updated definitions of IFDs should prove applicable in clinical, diagnostic, and epidemiologic research of a broader range of patients at high-risk.

## Teaser

European Organization for Research and Treatment of Cancer and the Mycoses Study Group definitions for probable invasive fungal disease now include solid organ transplant, hematologic malignancy, graft-versus-host disease, the reverse halo sign, revised thresholds for galactomannan, *Aspergillus* polymerase chain reaction, and definitions for candidiasis, cryptococcosis, non-human immunodeficiency viirus–associated pneumocystosis, and endemic mycoses.

**(See the Editorial Commentary by Rogers on pages 1377–8.)**

The European Organization for Research and Treatment of Cancer and the Mycoses Study Group Education and Research Consortium (EORTC/MSGERC) consensus definitions of invasive fungal diseases (IFDs) were last updated in 2008 [1]. These definitions achieved their original aim in fostering communication and enabling comparison of study findings among those engaged in research into IFD of patients with cancer and recipients of hematopoietic stem cell transplants (HSCTs) or solid organ transplants (SOTs) [2, 3]. Moreover, they have been adopted by regulatory agencies for evaluation of antifungals and have been used to evaluate diagnostic tests and to conduct epidemiologic studies [4–7]. Importantly, these definitions are specifically intended for these purposes only and not to direct or guide patient care.

The 2008 definitions had their shortcomings, including the facts that the definitions were unsuitable for patients with IFD in the setting of intensive care units (ICUs) or in pediatrics, data were insufficient to establish appropriate thresholds for detecting *Aspergillus* galactomannan (GM), and there was uncertainty about the role of (1,3)-beta-D glucan (BDG). Furthermore, nucleic acid amplification including polymerase chain reaction (PCR)–based tests were excluded because of lack of standardization and validation. Definitions for cryptococcosis and endemic mycoses also needed clarification, and there were no definitions for pneumocystosis.

## PROCESS

Volunteers from the EORTC Infectious Diseases Group and the MSGERC were assigned according to their expertise to 10 working groups, each charged with appraising a particular topic (see list of contributors in the Notes section). The chairs of the EORTC and MSG (J. P. D. and P. G. P.) selected leaders for each working group, and S. C. served as executive secretary. After completing the first round of working group assignments, leaders presented each group’s initial deliberations and recommendations at the 7th Trends in Medical Mycology Conference in Lisbon, Portugal, October 2015. A slide set was made available until 31 December 2015 online at www.e-materials.com/timm2015/invitation/Member and, on request, for public comment. After several iterations, the final draft of the manuscript was circulated to all members for their approval.

## REVISIONS AND UPDATES

### Special Populations

Pediatrics and patients in the ICU were considered as special populations. However, group 10 (IFD definitions in ICU patients) was unable to generate recommendations that preserved a level of certainty consistent with the existing definitions except for proven IFD (Table 1) and therefore undertook a separate initiative [8].

**Table 1.** Criteria for Proven Invasive Fungal Disease

Fungus	Microscopic Analysis: Sterile Material	Culture: Sterile Material	Blood	Serology	Tissue Nucleic Acid Diagnosis
Molds^a^	Histopathologic, cytopathologic, or direct microscopic examination^b^ of a specimen obtained by needle aspiration or biopsy in which hyphae or melanized yeast-like forms are seen accompanied by evidence of associated tissue damage	Recovery of a hyaline or pigmented mold by culture of a specimen obtained by a sterile procedure from a normally sterile and clinically or radiologically abnormal site consistent with an infectious disease process, excluding BAL fluid, a paranasal or mastoid sinus cavity specimen, and urine	Blood culture that yields a mold^c^ (eg, *Fusarium* species) in the context of a compatible infectious disease process	Not applicable	Amplification of fungal DNA by PCR combined with DNA sequencing when molds are seen in formalin-fixed paraffin-embedded tissue
Yeasts^a^	Histopathologic, cytopathologic, or direct microscopic examination of a specimen obtained by needle aspiration or biopsy from a normally sterile site (other than mucous membranes) showing yeast cells, for example, *Cryptococcus* species indicating encapsulated budding yeasts or *Candida* species showing pseudohyphae or true hyphae^d^	Recovery of a yeast by culture of a sample obtained by a sterile procedure (including a freshly placed [<24 hours ago] drain) from a normally sterile site showing a clinical or radiological abnormality consistent with an infectious disease process	Blood culture that yields yeast (eg, *Cryptococcus* or *Candida* species) or yeast-like fungi (eg, *Trichosporon* species)	Cryptococcal antigen in cerebrospinal fluid or blood confirms cryptococcosis	Amplification of fungal DNA by PCR combined with DNA sequencing when yeasts are seen in formalin-fixed paraffin-embedded tissue
Pneumocystis	Detection of the organism microscopically in tissue, BAL fluid, expectorated sputum using conventional or immunofluorescence staining	Not applicable	Not applicable	Not applicable	Not applicable
Endemic mycoses	Histopathology or direct microscopy of specimens obtained from an affected site showing the distinctive form of the fungus	Recovery by culture of the fungus from specimens from an affected site	Blood culture that yields the fungus	Not applicable	Not applicable

Abbreviations: BAL, bronchoalveolar lavage; PCR, polymerase chain reaction.

^a^If culture is available, append the identification at the genus or species level from the culture results.

^b^Tissue and cells submitted for histopathologic or cytopathologic studies should be stained using Grocott-Gomori methenamine silver stain or periodic acid Schiff stain to facilitate inspection of fungal structures. Whenever possible, wet mounts of specimens from foci related to invasive fungal disease should be stained with a fluorescent dye (eg, calcofluor or blankophor).

^c^Recovery of *Aspergillus* species from blood cultures rarely indicates endovascular disease and almost always represents contamination.

^d^ *Trichosporon* and yeast-like *Geotrichum* species and *Blastoschizomyces capitatus* may also form pseudohyphae or true hyphae.

#### Pediatrics: Group 1

There was a clear need to establish pediatric-specific IFD definitions as the clinical and radiologic manifestations of IFD in children, that is, patients aged <18 years, may differ significantly from those in adults. Most importantly, the incidence of invasive candidiasis (IC) is higher in neonates than in other age groups [9–11]. The degree of prematurity, based on gestational age and birth weight, is a unique risk factor among neonates; hematogenous *Candida* meningoencephalitis affects premature infants disproportionately and has serious consequences including seizures, intraventricular hemorrhage, and developmental delay [12, 13]. With respect to IC due to non-*Candida albicans* species, *Candida glabrata* is the most common pathogen in adults, whereas *Candida parapsilosis* predominates in children and neonates [14]. Risk factors for invasive mold diseases include innate immunologic defects, with *Aspergillus nidulans* being associated with chronic granulomatous disease, while *Aspergillus fumigatus* is seen more often in other patient groups [15].

Neonates with IC often present with subtle clinical findings, and cultures are frequently sterile, including cerebrospinal fluid (CSF) samples from neonates with candidemia and concurrent *Candida* meningitis. Diagnosis is often inferred from insensitive and nonspecific surrogate tests, such as increased C-reactive protein or thrombocytopenia, which has been shown to be a predictor of candidemia in infants [16, 17]. In neonates, a positive urine culture has a significance similar to that of a positive blood culture as an indicator of IC [16]. Radiographic findings are less specific in children than those reported in adults [18]. Chest computerized tomography (CT) scans in children with proven invasive pulmonary aspergillosis (IPA) commonly show nonspecific changes and not the halo sign, air crescent formation, or cavitation seen in adults [19].

There are also far fewer data to support the clinical use of nonculture-based fungal biomarkers in neonates and children [20], although the GM assay performs similarly in children and adults when used as an adjunctive tool to diagnose invasive aspergillosis (IA) [20, 21]. Likewise, there are few data regarding the use of BDG, *Candida* mannan antigen, and anti-mannan antibody biomarkers in pediatrics [22]. Recent data support the utility of BDG in CSF for the diagnosis and therapeutic monitoring of children with *Candida* meningoencephalitis [23], but the data are sparse regarding the utility of PCR assays and the T2Candida assay for diagnosis [24].

### Diagnostic Tests and Imaging

In the previous definitions [1], indirect tests for diagnosing IFD were only included if there was sufficient evidence that they had been standardized and validated. Moreover, commercial tests were included only if criteria for interpretation had been provided. Hence, while tests for GM and BDG were incorporated, tests for detecting fungal nucleic acid were not [1]. Furthermore, there was no agreement about appropriate thresholds, so the manufacturers’ analytical thresholds were adopted. The evidence for using GM to diagnose IA has grown considerably since then, and testing for BDG has been extended to a wide range of patients. With respect to *Aspergillus* PCR, the International Society of Human and Animal Mycology working group Fungal PCR Initiative (FPCRI; www.fpcri.eu) has made significant progress toward setting a standard for the technique after vigorous validation [25].

#### Imaging: Group 2

There is mounting evidence that the radiologic manifestations of invasive mold disease are more varied than previously recognized. The increased sensitivity of newer imaging techniques enables a greater number and depth of abnormalities to be seen in different anatomic regions. Recent data relating to the role of imaging in the diagnosis of IPA and pulmonary mucormycosis (PM) in adults suggest that a high-resolution CT scan (HRCT) is preferred to chest radiographs, magnetic resonance imaging (MRI), and positron emission tomography (PET), likely reflecting that HRCT is more sensitive than a chest radiograph, more widely available than MRI, and the experience with HRCT is much larger than with PET [26, 27]. Among patients with IPA, nodules or infiltrates with a halo sign remain useful among neutropenic patients but they are nonspecific for IPA in other groups [28]. Furthermore, the air crescent sign is a late and nonspecific sign. Among nonneutropenic patients, multiple pulmonary nodules and various nonspecific findings including bronchopneumonia, consolidation, cavitation, pleural effusions, ground glass opacities, tree-in-bud opacities, and atelectasis are found [29]. Overall, consolidation is the most frequent presentation of PM, followed by mass lesions, nodules, and cavitation [30]. Multiple nodules (more than10) and pleural effusions appear to be more frequent in PM than in IPA [31]. Moreover, the reverse halo sign is more specific for PM than IPA, although the differential diagnosis also includes other diseases including tuberculosis [32].

#### Aspergillus Galactomannan: Group 3

We evaluated *Aspergillus* galactomannan for both adults and children and specific patient groups and its utility and validity for different clinical specimens. We adopted different thresholds for different specimens rather than for different host groups [33–35] (Table 2). These differ from those recommended by the manufacturer of the GM assay (Platelia *Aspergillus* (Bio-Rad, CA), validated only for use in serum and bronchoalveolar lavage (BAL) fluid; however, detection of GM in plasma and CSF should support a diagnosis of IA [36, 37]. Exposure to mold-active antifungals compromises the utility of the GM test for IA [38] by reducing its sensitivity [39]. Therefore, caution should be exercised when GM is found to be absent from serum or plasma in patients receiving mold-active antifungals. There was consensus that similar GM thresholds are appropriate for adults and children.

**Table 2.** Probable Invasive Pulmonary Mold Diseases

**Host factors**
Recent history of neutropenia (<0.5 × 10^9^ neutrophils/L [<500 neutrophils/mm^3^] for >10 days) temporally related to the onset of invasive fungal disease
Hematologic malignancy^a^
Receipt of an allogeneic stem cell transplant
Receipt of a solid organ transplant
Prolonged use of corticosteroids (excluding among patients with allergic bronchopulmonary aspergillosis) at a therapeutic dose of ≥0.3 mg/kg corticosteroids for ≥3 weeks in the past 60 days
Treatment with other recognized T-cell immunosuppressants, such as calcineurin inhibitors, tumor necrosis factor-a blockers, lymphocyte-specific monoclonal antibodies, immunosuppressive nucleoside analogues during the past 90 days
Treatment with recognized B-cell immunosuppressants, such as Bruton’s tyrosine kinase inhibitors, eg, ibrutinib
Inherited severe immunodeficiency (such as chronic granulomatous disease, STAT 3 deficiency, or severe combined immunodeficiency)
Acute graft-versus-host disease grade III or IV involving the gut, lungs, or liver that is refractory to first-line treatment with steroids
**Clinical features**
*Pulmonary aspergillosis*
The presence of 1 of the following 4 patterns on CT:
Dense, well-circumscribed lesions(s) with or without a halo sign
Air crescent sign
Cavity
Wedge-shaped and segmental or lobar consolidation
*Other pulmonary mold diseases*
As for pulmonary aspergillosis but also including a reverse halo sign
*Tracheobronchitis*
Tracheobronchial ulceration, nodule, pseudomembrane, plaque, or eschar seen on bronchoscopic analysis
*Sino-nasal diseases*
Acute localized pain (including pain radiating to the eye)
Nasal ulcer with black eschar
Extension from the paranasal sinus across bony barriers, including into the orbit
*Central nervous system infection*
1 of the following 2 signs:
Focal lesions on imaging
Meningeal enhancement on magnetic resonance imaging or CT
**Mycological evidence**
Any mold, for example, *Aspergillus*, *Fusarium*, *Scedosporium* species or Mucorales recovered by culture from sputum, BAL, bronchial brush, or aspirate
Microscopical detection of fungal elements in sputum, BAL, bronchial brush, or aspirate indicating a mold
*Tracheobronchitis*
*Aspergillus* recovered by culture of BAL or bronchial brush
Microscopic detection of fungal elements in BAL or bronchial brush indicating a mold
*Sino-nasal diseases*
Mold recovered by culture of sinus aspirate samples
Microscopic detection of fungal elements in sinus aspirate samples indicating a mold
*Aspergillosis only*
*Galactomannan antigen*
Antigen detected in plasma, serum, BAL, or CSF
Any 1 of the following:
Single serum or plasma: ≥1.0
BAL fluid: ≥1.0
Single serum or plasma: ≥0.7 and BAL fluid ≥0.8
CSF: ≥1.0
*Aspergillus PCR*
Any 1 of the following:
Plasma, serum, or whole blood 2 or more consecutive PCR tests positive
BAL fluid 2 or more duplicate PCR tests positive
At least 1 PCR test positive in plasma, serum, or whole blood and 1 PCR test positive in BAL fluid
*Aspergillus* species recovered by culture from sputum, BAL, bronchial brush, or aspirate

Probable invasive fungal diseases (IFD) requires the presence of at least 1 host factor, a clinical feature and mycologic evidence and is proposed for immunocompromised patients only, whereas proven IFD can apply to any patient, regardless of whether the patient is immunocompromised. Probable IFD requires the presence of a host factor, a clinical feature, and mycologic evidence. Cases that meet the criteria for a host factor and a clinical feature but for which mycological evidence has not been found are considered possible IFD. (1,3)-beta-D glucan was not considered to provide mycological evidence of any invasive mold disease.

Abbreviations: BAL, bronchoalveolar lavage; CSF, cerebrospinal fluid; CT, computed tomography; PCR, polymerase chain reaction.

^a^Hematologic malignancy refers to active malignancy, in receipt of treatment for this malignancy, and those in remission in the recent past. These patients would comprise largely acute leukemias and lymphomas, as well as multiple myeloma, whereas patients with aplastic anemia represent a more heterogeneous group of individuals and are not included.

#### BDG and T2Candida Assays: Group 4

The group considers detection of BDG to be suitable for diagnosing probable IFD in the appropriate clinical setting. This includes patients with hematologic malignancies with and without neutropenia, neutropenia following HSCT, and certain patients in the ICU who are at higher risk (>10%) for IC as a result of gastrointestinal surgery with recurrent anastomotic leaks, perforations of the upper gastrointestinal tract, or necrotizing pancreatitis when there is clinical suspicion of infection [40, 41]. A single threshold (>80 pg/mL) using the Fungitell test (Associates of Cape Cod, Falmouth, MA) is recommended; there is insufficient evidence to include assays produced by other manufacturers [42]. Confidence for true positive results increases with repeated positive tests and for values that greatly exceed the positivity threshold [43]. There may be variability in positive predictive value (PPV) and negative predictive value (NPV) based on patient population, but a single threshold is favored at this time. The group did not support the use of serum BDG to rule in patients for clinical trials or for defining IA or IC, as BDG detection is not specific for any one IFD. It was agreed that this test should only be used on serum samples, although the test has been used for CSF samples with some success to support a diagnosis of central nervous system (CNS) IFD in certain circumstances when other diagnostic tests are negative or inconclusive [44].

The T2Candida panel has been cleared by the US Food and Drug Administration for the detection of common *Candida* species from whole blood specimens. The test has a very high NPV but, as with all such tests, the PPV is variable and depends upon disease prevalence in a given patient population [45, 46]. The PPV increases from 62% among patients with sepsis, shock, or lengths of stay greater than 3–7 days in an ICU to 92% for bone marrow transplant recipients and patients with leukemia who are neutropenic but not receiving any antifungal prophylaxis. The test has been included as mycologic evidence to support a diagnosis of candidemia in selected clinical trials [47].

#### Aspergillus PCR: Group 5

In considering *Aspergillus* PCR, target species, patient populations, appropriate specimens for testing, technical issues, comparison with other biomarker assays, and unique attributes of PCR assays were reviewed. The data were sufficiently robust for performing *Aspergillus* PCR on serum, plasma, whole blood, and BAL fluid in adults. The group acknowledged that *Aspergillus* PCR data have been evaluated most extensively for adults with hematologic malignancies and HSCT. Systematic reviews of *Aspergillus* PCR methods on blood and BAL fluid conclude that PCR provides a robust diagnostic test for screening and confirming the diagnosis of *Aspergillus* infection [22, 48–53].

There are relatively few commercial PCR assays, and most investigators have developed methods in-house. As such, the FPCRI was established to develop criteria for *Aspergillus* PCR rather than a standardized method per se. Despite technologic variability, PCR performance was comparable with that for detecting GM and BDG [54]. Moreover, commercial PCR tests performed using methodology in line with the FPCRI recommendations provide a standardized approach that has been independently associated with improved performance. A unique feature of PCR is its ability to detect both genus and species of *Aspergillus*. PCR is also capable of identifying certain mutations associated with triazole resistance directly from clinical specimens [55–57].

#### Tissue Diagnosis: Group 6

Tissue diagnosis requires the presence of fungal elements in formalin-fixed paraffin-embedded tissue and signifies proven fungal disease but not the identity of the fungus involved. To achieve this, we recommend amplification of fungal DNA by PCR combined with DNA sequencing, but only when fungal elements are seen by histopathology. PCR would add value by allowing identification of the fungus to genus and possibly species levels. Because the technique used should be rigorously quality controlled, only laboratories with a proven record in performing DNA extraction from formalin-fixed tissue should undertake this. The identity of the fungus should be consistent with the histopathologic findings [58–60].

### Other Disease Entities

#### Pneumocystosis: Group 7

The inclusion of *Pneumocystis jirovecii* pneumonia (PCP) diagnosis in the updated definitions was limited to patients not living with human immunodeficiency virus (HIV). Diagnosing PCP has been more difficult among these patients possibly due to a more focal pulmonary involvement, lower suspicion of disease, and lower sensitivity of traditional histologic and microscopy diagnostic tests [61]. As such, it is important to more fully define host factors for patients at increased risk for PCP. We selected receipt of therapeutic doses of corticosteroids for at least 2 weeks within the past 60 days; antineoplastic, antiinflammatory, or immunosuppressive treatment; and low CD4 lymphocyte counts due to a medical condition. This includes, but is not limited to, patients with primary immunodeficiencies, hematologic malignancies, SOTs, and allogeneic HSCT recipients [62, 63]. Clinical criteria in this population tend to be nonspecific and include cough, dyspnea, and hypoxemia. Radiographic abnormalities include bilateral ground-glass opacities and, less frequently, consolidation, small nodules, unilateral infiltrates, pleural effusions, and cystic lesions [61, 64, 65]. Amplification of *P. jirovecii* DNA by quantitative real-time PCR on BAL fluid, expectorated sputum, or oral wash specimens is preferred to qualitative PCR and is helpful to establish probable disease. However, further studies are needed to validate thresholds for positivity [66, 67]. Similarly, 2 or more serum BDG levels of ≥80 ng/L are useful for diagnosing probable disease in appropriate clinical context provided other IFDs have been excluded [68, 69].

#### Cryptococcosis: Group 8

A broader understanding of the natural history and host factors associated with cryptococcal disease warrants updating these definitions. We support the previous definitions of proven and probable cryptococcal disease in any host. However, we also recognize cryptococcal infection among individuals in high-risk host groups who have few, if any, symptoms and only a positive serum cryptococcal antigen test (asymptomatic cryptococcal antigenemia). This condition may be more common than symptomatic disease, and patients may develop clinical cryptococcal disease unless treated and so are now included in these definitions [70]. The term “disseminated cryptococcosis” as distinct from CNS cryptococcosis has been abandoned in favor of the terms “pulmonary,” “CNS” and “other extrapulmonary sites.” “Colonization” with *Cryptococcus* spp. is no longer included in the definitions as it is poorly understood and its natural history is unknown.

Identification to the species level for *Cryptococcus neoformans* and *Cryptococcus gattii* has become increasingly important based on reports that suggest different clinical presentations, outcomes, and responses to antifungal therapy between these 2 species [71, 72]. Verification of species that use CGB (L-canavanine, glycine, bromthymol blue) agar or matrix-assisted laser desorption ionization–time of flight mass spectrometry is recommended. Outcomes for HIV-associated cryptococcosis due to *C. neoformans* and *C. gattii* appear to be similar, and identification to the species level may be unnecessary [73, 74].

#### Endemic Mycoses: Group 9

The endemic mycoses are caused by environmental fungi that are usually restricted geographically and cause disease in immunocompetent and immunocompromised hosts. Causative agents include *Histoplasma capsulatum* var. *capsulatum* and *H. capsulatum* var. *duboisii*, *Blastomyces* species complex (eg, *B. dermatitidis*, *B. gilchristii*, *B. helicus*, *B. silverae*, and *B. parvus*), *Coccidioides immitis/Coccidioides posadasii*, *Paracoccidioides brasiliensis/Paracoccidioides lutzii, Sporothrix* species complex (*S. brasiliensis*, *S. schenckii sensu stricto*, *S. globosa*, and *S. luriei*), *Talaromyces* (formerly *Penicillium*) *marneffei*, and *Emergomyces* species (*E. pasteurianus*, *E. africanus*, *E. orientalis*, *E. canadensis*, and * E. europaeus*) [75–80]. Probable endemic mycoses are defined by evidence of environmental exposure to the fungus, a compatible clinical illness, and the presence of either *Histoplasma* or *Blastomyces* antigen in any body fluid or antibody to *Coccidioides* species in serum or CSF as recovery by culture and histopathologic evidence of infection is generally lacking. There are no approved serologic tests for *T. marneffei*, *S. schenckii* species complex, or *P. brasiliensis*. Exposure to 1 of these fungi is defined as a history of residence in an endemic area, no matter how remote, travel to an endemic area, or contact with fomites such as soil or vegetation that is derived from an endemic area.

### Proven Invasive Fungal Disease

The revised definitions of proven IFD are shown in Table 1.

### Probable Invasive Fungal Disease

Several changes were made to the definitions of probable IFD (Tables 2 and 3). For example, host factors were expanded to include inherited severe immunodeficiency and low CD4 lymphocyte counts. Radiographic features were expanded to include wedge-shaped and segmental or lobar consolidation and a reverse halo sign to indicate mold disease of the lower respiratory tract. Revised thresholds for GM now replace those of the manufacturer. *Aspergillus* PCR is now included, and there are mycologic criteria for non-HIV–associated pneumocystosis.

**Table 3.** Other Probable Invasive Diseases

**Candidiasis**
*Host factors*
Recent history of neutropenia <0.5 × 10^9^ neutrophils/L (<500 neutrophils/mm^3^ for >10 days) temporally related to the onset of invasive fungal disease
Hematologic malignancy
Receipt of an allogeneic stem cell transplant
Solid organ transplant recipient
Prolonged use of corticosteroids (excluding among patients with allergic bronchopulmonary aspergillosis) at a therapeutic dose of ≥0.3 mg/kg corticosteroids for ≥3 weeks in the past 60 days
Treatment with other recognized T-cell immunosuppressants, such as calcineurin inhibitors, tumor necrosis factor-a blockers, lymphocyte-specific monoclonal antibodies, immunosuppressive nucleoside analogues during the past 90 days
Inherited severe immunodeficiency (such as chronic granulomatous disease, STAT 3 deficiency, CARD9 deficiency, STAT-1 gain of function, or severe combined immunodeficiency)
Acute graft-versus-host disease grade III or IV involving the gut, lungs, or liver that is refractory to first-line treatment with steroids
*Clinical features*
At least 1 of the following 2 entities after an episode of candidemia within the previous 2 weeks:
Small, target-like abscesses in liver or spleen (bull’s-eye lesions) or in the brain, or, meningeal enhancement
Progressive retinal exudates or vitreal opacities on ophthalmologic examination
*Mycological evidence*
ß-D-glucan (Fungitell) ≥80 ng/L (pg/mL) detected in at least 2 consecutive serum samples provided that other etiologies have been excluded
Positive T2Candida^a^
**Cryptococcosis**
*Host factors* ^b^
Human immunodeficiency virus infection
Solid organ or stem cell transplant recipient
Hematologic malignancy
Antibody deficiency (eg, common variable immunoglobulin deficiency)
Immunosuppressive therapy (including monoclonal antibodies)
End-stage liver or renal disease
Idiopathic CD4 lymphocytopenia
*Clinical features*
Meningeal inflammation
Radiological lesion consistent with cryptococcal disease
*Mycological evidence*
Recovery of *Cryptococcus* from a specimen obtained from any nonsterile site
**Pneumocystosis** ^**c**^
*Host factors*
Low CD4 lymphocyte counts <200 cells/mm^3^ (200 × 10^6^ cells/L) for any reason
Exposure to medication (antineoplastic therapy, antiinflammatory, or immunosuppressive treatment) associated with T-cell dysfunction
Use of therapeutic doses of ≥0.3 mg/kg prednisone equivalent for ≥2 weeks in the past 60 days
Solid organ transplant
*Clinical features*
Any consistent radiographic features particularly bilateral ground glass opacities, consolidations, small nodules or unilateral infiltrates lobar infiltrate, nodular infiltrate with or without cavitation, multifocal infiltrates, miliary pattern ^d^
Respiratory symptoms with cough, dyspnea, and hypoxemia accompanying radiographic abnormalities including consolidations, small nodules, unilateral infiltrates, pleural effusions, or cystic lesions on chest X-ray or computed tomography scan
*Mycological evidence*
ß-D-glucan (Fungitell) ≥80 ng/L (pg/mL) detection in ≥2 consecutive serum samples provided other etiologies have been excluded
Detection of *Pneumocystis jirovecii* DNA by quantitative real-time polymerase chain reaction in a respiratory tract specimen
**Endemic mycoses**
*Host factors*
Not applicable as these diseases affect both healthy and less healthy hosts
*Clinical features*
Evidence for geographical or occupational exposure (including remote) to the fungus and compatible clinical illness
Mycological evidence
*Histoplasma* or *Blastomyces* antigen in urine, serum, or body fluid
Antibody to *Coccidioides* in cerebrospinal fluid or 2-fold rise in 2 consecutive serum samples

Probable invasive fungal diseases (IFD)requires the presence of at least 1 host factor, a clinical feature and mycologic evidence and is proposed for immunocompromised patients only, whereas proven invasive fungal disease can apply to any patient, regardless of whether the patient is immunocompromised. Except for endemic mycoses, probable IFD requires the presence of a host factor, a clinical feature, and mycologic evidence, whereas cases that meet the criteria for a host factor and a clinical feature but for which mycological evidence has not been found are considered possible IFD.

^a^T2Candida is US Food and Drug Administration approved for the detection of *Candida albicans*, *Candida parapsilosis*, *Candida tropicalis*, *Candida krusei,* and *Candida glabrata* in blood.

^b^Cryptococcosis also occurs in phenotypically normal hosts.

^c^Definitions for human immunodeficiency virus–associated pneumocystosis are not included here.

^d^Bilateral, diffuse ground glass opacities with interstitial infiltrates are more common than other features such as consolidations, small nodules, thin-walled cavities, and unilateral infiltrates.

### Possible Invasive Fungal Disease

While definitions of proven and probable disease have been shown to be reliable in research and attracted little controversy among the group, this cannot be said of the possible IFD category. There is much confusion about the difference between a host factor and a risk factor. As before, a host factor has been defined as a characteristic of individuals clearly predisposed to, and not simply at risk of, an IFD [1]. For example, while impaired gut wall integrity through surgery or illness may increase the risk of candidiasis, it was not considered specific enough to warrant inclusion as a host factor. Pulmonary abnormalities such as tree-in-bud opacities and interstitial abnormalities were excluded from the clinical features as they can be due to a wide range of pathologies in addition to IFD.

## GENERAL POINTS

Throughout this process, we have emphasized the need to differentiate between definitions of IFD required for clinical research from those that influence clinical practice. In clinical practice, many would administer an antifungal agent to any patient at risk of IFD when fungi are detected by biomarkers in serum, plasma, whole blood, or relevant body site fluid without there being sufficient evidence to satisfy the consensus definitions of IFD. We also recognize that our definition of a host factor errs on the side of conservatism given the increasing use of drugs such as monoclonal antibodies for treating a variety of conditions.

Other controversial issues included distinguishing between the performance characteristics of tests for screening and confirmation, the impact of exposure to antifungal agents used for prophylaxis or treatment on imaging and diagnostic tests, and the use of biomarkers to monitor therapeutic outcome. We agree that further research will be required to evaluate the evidence for each of these assays. Finally, there was consensus that diagnostic strategies to determine the relative efficiency of an available test, alone or in combination with other diagnostic tests, should be considered further.

## CONCLUSIONS

In summary, these revised definitions represent consensus expert opinion based on the best available evidence. As such, they will need to be reviewed regularly for their utility and relevance and, where possible, extended to other populations affected by IFDs. We acknowledge the limitations of these definitions, including the exclusion of certain cases of IFD. However, the reliance on host factors, clinical features, and mycologic evidence to define IFD in selected populations has proven its value for clinical trials, epidemiologic studies, and the evaluation of diagnostic tests.

## Notes

***Author contributions.*** *Pediatrics:* William Steinbach (Chair, Mycoses Study Group [MSG]), Brian Fisher (MSG), Andreas Groll (European Organization for Research and Treatment of Cancer [EORTC]) Thomas Lehrnbecher (EORTC), Emmanuel Roilides (EORTC), Thomas J Walsh (MSG), Adilia Warris (EORTC), Theo Zaoutis (MSG). *Guidance on imaging:* John W. Baddley (Chair, MSG), Barbara Alexander (MSG), Sujal Desai (EORTC), Klaus Peter Heussel (EORTC), Frédéric Lamoth (EORTC), Orla Morrisey (MSG), Cornelia Schaefer Prokop (EORTC). *Update of galactomannan:* Paul Verweij (Chair, EORTC), Elio Castagnola (EORTC), Johan Maertens (EORTC), Kieren Marr (MSG), Joseph Wheat (MSG). *Update on beta-D-glucan, T2Candida:* Cornelius J. Clancy (Chair, MSG), Hamdi Akan (EORTC), David Andes (MSG), Mario Cruciani (EORTC), Frédéric Lamoth (EORTC), Oscar Marchetti (EORTC), Luis Ostrosky- Zeichner (MSG), Zdenek Racil (EORTC). *Update of polymerase chain reaction:* John R. Wingard (Chair, MSG), Stephane Bretagne (EORTC), Angela Caliendo (MSG), Jurgen Loeffler (EORTC), Tom Patterson (MSG), Monica Slavin (MSG), P Lewis White (EORTC). *Tissue diagnosis:* Shawn Lockhart (Chair, MSG), Ralf Bialek (EORTC), Manuel Cuenca (EORTC), Henrik Jensen (EORTC), Chris Kibbler (EORTC), Dimitrios Kontoyiannis (MSG). *Pneumocystosis:* Andreas Groll (Chair, EORTC), Sharon Chen (MSG), Catherine Decker (MSG), Katrien Lagrou (EORTC), Henry Masur (MSG), Livio Pagano (EORTC), Claudio Viscoli (EORTC). *Cryptococcosis:* Tania Sorrell (Chair, MSG), Peter Pappas (MSG), Tom Harrison (EORTC), Olivier Lortholary (EORTC), John Perfect (MSG). *Endemic mycosis:* Carol A. Kauffman (Chair, MSG), Robert W. Bradsher (MSG), Jacques F. Meis (EORTC), Marcio Nucci (MSG), David Stevens (MSG), George R Thompson (MSG). *Inclusion of patients in the intensive care unit:* Matteo Bassetti (Chair, EORTC), Elie Azoulay (EORTC), Thierry Calandra (EORTC), Bart-Jan Kullberg (EORTC), Frédéric Lamoth (EORTC), Marcus Ruhnke (EORTC), Shmuel Shoham (MSG), Jose Vazquez (MSG).

***Potential conflicts of interest.*** H. Akan reports being a primary investigator in a clinical trial for MSD. B. D. Alexander reports grants from Astellas, Cidara, Scynexis, F2G, and Viamet and personal fees from Astellas, Scynexis, and UpToDate outside the submitted work. D. Andes reports grants and consulting fees from Amplyx, Matinas, Cidara, and Merck outside the submitted work. E. Azoulay reports personal fees from Gilead, Pfizer, Ablynx, Alexion, and Baxter; grants and personal fees from MSD; and conference invitation from Gilead outside the submitted work. M. Bassetti reports grants from Pfizer, MSD, and Cidara and personal fees from Pfizer, MSD, Astellas, ThermoFisher, Gilead, Cidara, Biomerieux, and Menarini outside the submitted work. S. Bretagne reports grants from MSD and Pfizer and personal fees from Gilead outside the submitted work. T. Calandra reports advisory board membership from Astellas, Basilea, Cidara, MSD, Sobi, ThermoFisher, and GE Healthcare and data monitoring board membership from Novartis. A. M. Caliendo reports grants from T2 Biosystems outside the submitted work. E. Castagnola reports personal fees from Gilead outside the submitted work. S. C. Chen reports grants from MSD Australia outside the submitted work. C. J. Clancy reports consultancy fees from Merck, Cidara, Astellas, The Medicine Company, Scynexis, and T2Biosystems; grants/pending grants from T2Biosystems, Merck, Cidara, and Astellas; and personal fees from Merck and T2 Biosystems outside the submitted work. M. Cuenca-Estrella reports grants and personal fees from Gilead Sciences and grants from F2G, Scynexis, Cidara, and Amplyx outside the submitted work. S. R. Desai reports paid honorarium and travel expenses from Interstitial Lung Disease MDT—Boehringer-Ingelheim. J. P. Donnelly reports personal fees from F2G Ltd, Gilead Sciences, and Pfizer outside the submitted work. B. Fisher reports grants from Pfizer, Merck, and the National Institutes of Health and personal fees from Astellas outside the submitted work. T. Harrison reports grants from Gilead Sciences and personal fees from Gilead Sciences, Pfizer, and Viamet outside the submitted work. C. P. Heussel reports personal fees from Basilea, Bayer, Boehringer-Ingelheim, Gilead, Interimmune, MSD, Novartis, and Pfizer; grants from the German Center for Lung Research, MeVis, Pfizer, and Siemens; and consultancy fees from Fresenius outside the submitted work. C. A. Kaufmann reports data safety monitoring board membership for Cidara Therapeudics outside the submitted work. D. P. Kontoyiannis reports personal fees from Merck & Co, Gilead Sciences, United Medical, Astellas Pharma, Pharma, Cidara Therapeutics, Amplyx Pharmaceuticals, and Mayne Pharma outside the submitted work. B.-J. Kullberg reports personal fees from Amplyx, Astellas, Cidara, Pfizer, and Scynexis outside the submitted work. K. Lagrou reports personal fees and travel support from Pfizer and MSD and personal fees from Abbott, SMB Laboratoires Brussels, Gilead, and Roche outside the submitted work. F. Lamoth reports personal fees from MSD and Basilea outside the submitted work. T. Lehrnbecher reports grants, personal fees, and nonfinancial support from Gilead Sciences; personal fees and nonfinancial support from MSD/Merck and Astellas; and personal fees from Basilea outside the submitted work. J. Maertens reports personal fees and nonfinancial support from Basilea Pharmaceuticals, Bio-Rad Laboratories, Cidara, F2G Ltd, Gilead Sciences, Merck, and Pfizer Inc and grants from Gilead Sciences outside the submitted work. K. A. Marr reports personal fees from Amplyx, Cidara, and Merck; licensing royalties from MycoMed Technologies; and patents from MycoMed outside the submitted work. J. F. Meis reports grants from F2G Ltd, personal fees from Gilead Sciences, other from Pulmocide, grants and personal fees from Scynexis, personal fees and other from TEVA, and personal fees and other from United Medical outside the submitted work. C. O. Morrissey reports grants from Gilead Sciences, Merck, Sharp and Dohme and advisory board membership from Merck, Sharp, and Dohme outside the submitted work. M. Nucci reports personal fees from Pfizer, MSD, Basilea, Gilead, Biotoscana, Teva, Abbvie, Astellas, and Janssen outside the submitted work. L. Ostrosky-Zeichner reports personal fees from Merck, F2G, Mayne, Viracor, and Gilead; grants and personal fees from Pfizer, Astellas, Scynexis, and Cidara; grants from Amplyx; and personal fees from Realtime outside the submitted work. L. Pagano reports personal fees from MSD and Pfizer and grants and personal fees from Gilead outside the submitted work. T. F. Patterson reports personal fees from Basilea; grants from Cidara; and personal fees from Gilead, Merck, Scynexis, Toyama, and Pfizer outside the submitted work. J. R. Perfect reports grants from Pfizer and Mayne; grants and other from Merck, Minnetronix, and Amplyx and other from Viamet, F2G, Vical, Matinas, Cidara during the conduct of the study. E. Roilides reports grants from Astellas Pharma, Gilead Sciences, MSD Sharp & Dohme, Pfizer outside the submitted work. M. Ruhnke reports personal fees from SCYNEXIS, Basilea, Kedplasma, and Daiichi outside the submitted work. S. Shoham reports grants from Merck, Astellas, Shionogi, Cidara, Scynexis, Shire, T2 Microsystems, Ansun, Emergent, and Gilead and personal fees from Merck outside the submitted work. M.A. Slavin reports grants from Merck; personal fees from Merck; and other from Pfizer and Gilead outside the submitted work. D. A. Stevens reports grants from Astellas and Riptide and consulting fees from Pulmatrix, the US Department of Justice, and Fresenius outside the submitted work. G. R. Thompson reports grants and other from Astellas, Cidara, Vical, Scynexis, F2G, and Mayne and grants from RealTime Labs outside the submitted work. J. A. Vazquez reports personal fees from Astellas, Cidara, Amplyx, and F2G outside the submitted work. C. Viscoli reports personal fees from MSD Int, Gilead, Pfizer, Angelini, Astellas, and Basilea outside the submitted work. P. E.Verweij reports grants from Gilead Sciences, MSD, Pfizer, and F2G and nonfinancial support from OLM and IMMY outside the submitted work. T. J. Walsh has received grants for experimental and clinical antifungal pharmacology and molecular diagnostics to his institution from Amplyx, Astellas, Lediant, Merck, Scynexis, and T2 Biosystems and has served as a consultant to Amplyx, Astellas, Gilead, Lediant, Merck, and Scynexis. A. Warris reports grants and personal fees from Gilead outside the submitted work. P. L. White reports nonfinancial support from Bruker, Dynamiker, and Launch; personal fees and nonfinancial support from Gilead; and personal fees from MSD outside the submitted work. J. R. Wingard reports personal fees from Ansun, Astellas, Behring, Celgene, Cidara, Merck, and Shire outside the submitted work. T. E. Zaoutis reports personal fees from Pfizer outside the submitted work. All other authors report no potential conflicts. All authors have submitted the ICMJE Form for Disclosure of Potential Conflicts of Interest. Conflicts that the editors consider relevant to the content of the manuscript have been disclosed.
